# Educational

**Published:** 1887-10

**Authors:** 


### EDITORIAL.

### Educational.

The period for the beginning of all educational institutions throughout our country is near at hand. October 1st is the time at which nearly all medical and dental colleges begin their regular course of instruction. Students are active in their preparation for entering upon their college work. So far at least as dental students are concerned the probability is that by far the larger part have already selected the institution in which they will enter.

In entering upon a course of dental study and work, various motives are operative with different individuals. A considerable number—we will not venture an opinion as to what per-cent—are influenced in the main by material, or, perhaps more definitely, commercial consideration. Such students often remark: "I think dentistry is a good and profitable business," showing at once by such a remark that the commercial motive is the leading one. Others, again, of a little higher aspiration, perhaps not losing sight of this feature, are influenced withal by a higher motive, namely, that of engaging in an honorable occupation and and of being reckoned in public estimation as a member of a profession, thus aiming to gratify an ambition that within certain limits is quite commendable. Others, again, enter upon their work with a still higher motive, namely, that of adding to the well-being of their fellow-men. With a few this is the leading motive, but with a far larger proportion each of these motives has more or less influence, and when properly distributed make, upon the whole, the best arrangement. The desire to procure a competency of this world's good things, and also to have a name and reputation among one's fellows, are, in proper measure, not at all incompatible with the best and highest service to humanity.

All dentists who accept students and become in any wise responsible for their early instruction should not fail to give proper teaching on these ethical questions. They are quite as important as the more practical things that are regarded by some as making up the total of one's professional education. Those who enter college without private pupilage should, of course, in the institution they enter, receive the proper instruction upon these points. Indeed all the ethical and the common moral points that go to make up the true man should receive attention at the hands of the teacher, whether he be public or private. This need not involve the laying out of a complete moral and ethical code, but the teacher, always by example and by precept, when need be, should be found upon the right side. It is impossible for the teacher to estimate the influence his course of life, aside from that of his mere teaching, will have upon impressible minds. If the student is faulty in his habits, in any respect, in his course of life, it should be the business of his preceptor or teacher to show him both by example and by precept a better way. It is true he may not always succeed in bringing him into the better way, or to a higher standard, but there will be the consciousness of duty done, and the hope that at some favorable time the seed that may have been planted will spring up and bear fruit.

The obligations upon our dental teachers are becoming greater each year, and they are certainly now stronger than ever before. Dentistry, as a specialty of the healing art, has within the last few months been officially placed side by side with other departments of medical practice, and it is expected that the dental practitioner in the future will be all that the true medical man ought to be. This increases the responsibility of both the teacher and the taught, and the labor of each as well; and it is to be hoped that this will be fully recognized by all, and that the work of dental teaching and study this year will be entered upon with higher aims and aspirations than ever before.

